# Early-Onset Colorectal Cancer—A Retrospective Study from a Tertiary Referral Hospital in Romania

**DOI:** 10.3390/diagnostics14101052

**Published:** 2024-05-19

**Authors:** Elena Savu, Valeriu Șurlin, Liviu Vasile, Ileana Octavia Petrescu, Cristina Elena Singer, Nicolae-Daniel Pirici, Stelian Stefanita Mogoanta

**Affiliations:** 1 Doctoral School, University of Medicine and Pharmacy of Craiova, 200349 Craiova, Romania; 2 Department of Oncopediatrics, Clinical Emergency County Hospital, 200642 Craiova, Romania; 3 Department of General Surgery, Faculty of Medicine, University of Medicine and Pharmacy of Craiova, 200349 Craiova, Romania; valeriu.surlin@umfcv.ro; 4 First General Surgery Department, Clinical Emergency County Hospital, 200642 Craiova, Romania; 5 Department of Surgical Semiology, Faculty of Medicine, University of Medicine and Pharmacy of Craiova, 200349 Craiova, Romania; vliviu777@yahoo.com; 6 Third General Surgery Department, Clinical Emergency County Hospital, 200642 Craiova, Romania; ssmogo@yahoo.com; 7 Department of Pediatrics, Faculty of Medicine, University of Medicine and Pharmacy of Craiova, 200349 Craiova, Romania; petrescu.ileanaoctavia@yahoo.com (I.O.P.); singercristina@gmail.com (C.E.S.); 8 Second Pediatrics Department, Clinical Emergency County Hospital, 200642 Craiova, Romania; 9 Department of Histology, Faculty of Medicine, University of Medicine and Pharmacy of Craiova, 200349 Craiova, Romania; daniel.pirici@umfcv.ro; 10 Department of General Surgery, Faculty of Dental Medicine, University of Medicine and Pharmacy of Craiova, 200349 Craiova, Romania

**Keywords:** colorectal cancer, early-onset, screening, colorectal cancer symptoms, histopathology, polyps

## Abstract

Early-onset colorectal cancer emerges as a distinctive clinical and biological entity and is generally defined as the onset of colon or rectal neoplasia before the age of 50. Several reports describe an increasing incidence worldwide of colorectal cancers occurring in individuals younger than 50 years, along with particular histologic and molecular features. Although heredity may be an explanation in some cases with young-onset colorectal cancer, other driving factors remain partially unknown. The present study explores demographic, clinical, and pathological features within a group of patients diagnosed with colorectal cancer before the age of 50. It is a retrospective survey based on data collected between 2017 and 2023 within three surgical departments from a tertiary Romanian hospital. The clinical and pathological features we identified (later-stage disease, distal colon tumor localization, mucinous histology) are mainly superimposed with the existing data in the literature regarding this pathology. In order to lower the burden that colorectal neoplasia diagnosed in the young implies, a change of paradigm should be made in terms of establishing effective and targeted screening programs but also in the direction of enhancing complex clinical, pathological, and molecular diagnosis.

## 1. Introduction

The most recent data on the global cancer burden places colorectal cancer (CRC) among the top three cancer sites in terms of both incidence and mortality [1]. Furthermore, it is the leading cause of death in men under the age of 50 [2]. A worrisome trend was observed in the young population, where the occurrence of CRC has been steadily increasing over the last four decades, and projections anticipate a continuous rise for the next decade [3,4].

There is no standard and internationally accepted definition for early-onset colorectal cancer (EO-CRC), but considering that most of the screening programs commence at the age of 50, this cutoff has been chosen by most of the authors to define this entity [5,6].

The incidence of EO-CRC is rising across many developed regions worldwide, with some regional differences [7]. In Europe, the occurrence of colorectal cancer is increasing among individuals aged 20–49 years; of these, nearly three-quarters occur between 40 and 49 years [8,9].

The causes behind the rising incidence of EO-CRC are still not fully understood. Early-life exposure to various factors, including dietary, environmental, or chemical factors, triggers genetic and epigenetic alterations and might explain the increasing incidence of EO-CRC [10]. While the occurrence of colorectal cancer at a young age suggests a potential hereditary component, hereditary colorectal cancer syndromes account for only a small percent of CRC cases in the young population [11].

The burden of CRC may be reduced through the effective deployment of screening strategies in general and targeted populations [12]. While the United States Preventive Services Task Force advocates for lowering the starting age for screening from 50 to 45 years [13], CRC screening programs across Europe exhibit significant disparities, encompassing organized and opportunistic programs, various testing methods, and different rates of participation and detection [14].

EO-CRC appears to exhibit distinct characteristics compared to CRC in elderly individuals. Typically, EO-CRC manifests with more aggressive traits, is identified at later stages, and demonstrates a stronger potential for metastasis [9,15,16]. Conversely, young individuals diagnosed with metastatic cancer tend to experience superior overall survival rates, likely due to their improved performance status, fewer comorbidities, greater tolerance to chemotherapy regimens, and reduced risk of postoperative mortality [17].

There is a scarcity of nationwide studies addressing the issue of colorectal cancer in the Romanian young population. The present study aimed to analyze the incidence trends of EO-CRC within a population from a region in Romania during the study period to confirm or invalidate the documented incidence trends observed globally. Additionally, it aimed to perform an analysis of clinical and pathological parameters in the selected population of patients. To our knowledge, this is the first study from the specified Romanian region that provides a snapshot of the current status of EO-CRC and potentially serves as a basis for further comparative studies.

## 2. Materials and Methods

We conducted a retrospective, observational study in order to identify patients younger than 50 years diagnosed with colorectal cancer from three surgical departments in the Clinical Emergency County Hospital Craiova, Romania, between January 2017 and December 2023. The study included patients from the entire Oltenia region, from Dolj, Olt, Valcea, Mehedinti, and Gorj counties.

Relevant data were selected from the hospital’s electronic database and paper records. The inclusion criteria were age between 18 and 49 years, a confirmed diagnosis of colorectal cancer based on the pathological reports, and primary colon or rectal neoplasia. Malignant epithelial and mesenchymal tumors of the colon and rectum, according to WHO classification [18], were included. Patients with benign tumors, colorectal lymphoma, or secondary colon or rectal cancer were excluded. Metachronous cancer was considered if another tumor was identified after 1 year or more after resection of the primary lesion and located away from the suture line. Synchronous lesions were defined by the presence of another lesion away from the primary tumor at the time of the diagnosis or within 1 year.

The collected data included demographic aspects (age, gender, area of origin), clinical aspects (tumor localization, type of presentation, presence of tumor complication, comorbidities), and pathological data according to the pathological report. Tumor staging was assigned according to the TNM Classification of Malignant Tumours, eighth edition [19]. The anatomic sub-sites of the colon (left, right) and rectum were categorized according to the International Classification of Diseases for Oncology, third edition [20] topography codes. Right-sided colon cancers were identified using the following cancer site codes: cecum (code C18.0), ascending colon (code C18.2), hepatic flexure (code C18.3), and transverse colon (code C18.4). Left-sided colon cancers were identified using the codes splenic flexure (code C18.5), descending colon (code C18.6), sigmoid colon (code C18.7), and rectosigmoid (code C19.9). Rectal cancer was identified as code C20.

All the patients’ data were organized and consequently analyzed using descriptive statistics using Microsoft Excel 2019 MSO (version 2304 Build 16.0.16327.20200).

The study was conducted after the approval of the Ethics Committee of the Clinical Emergency County Hospital Craiova.

## 3. Results

**Figure 1:**
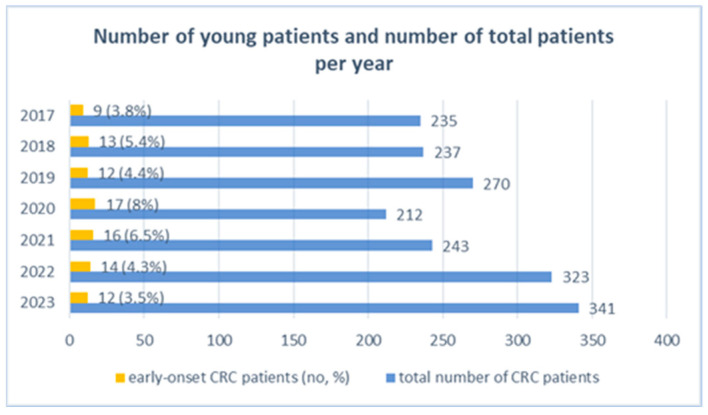
Histogram reflecting the number and percentage of EO-CRC patients from the total patients with CRC admitted each year.

**Figure 2:**
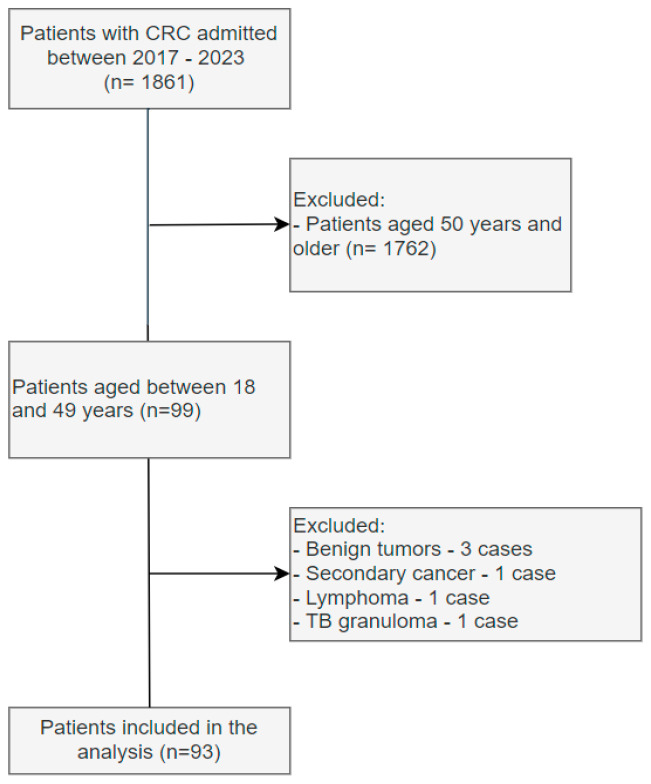
Flow diagram of the patient selection process.

The total number of patients with a colorectal cancer diagnosis admitted to the surgical departments regardless of age throughout the study period (2017–2023) was 1861 patients. Of these, 5% (93 cases) represented the patients with early-onset colorectal cancer and were included in the final analysis (Figure 1 and Figure 2).

**Figure 3:**
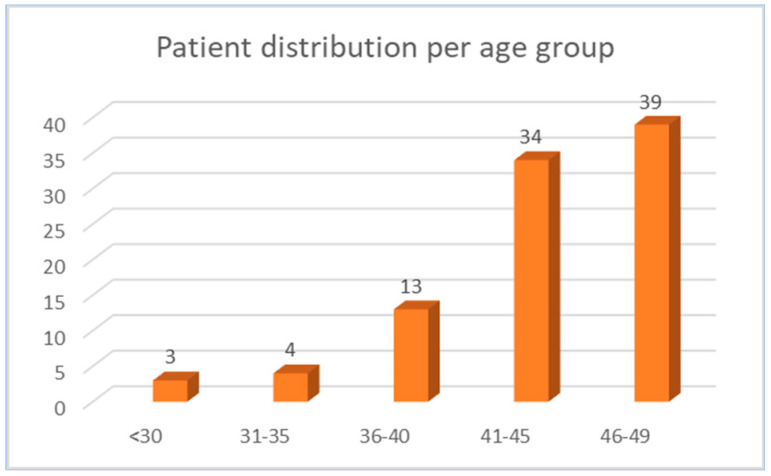
Age group distribution of patients with EO-CRC.

When analyzing the distribution of patients according to age, five age intervals were chosen (below 30 years, 31–35 years, 36–40 years, 41–45 years, and 46–49 years). A significant increase in the number of cases was noted after the age of 40, with a slight increase after the age of 46. However, the incidence trend noticeably increased after the age of 35 (Figure 3).

**Figure 4:**
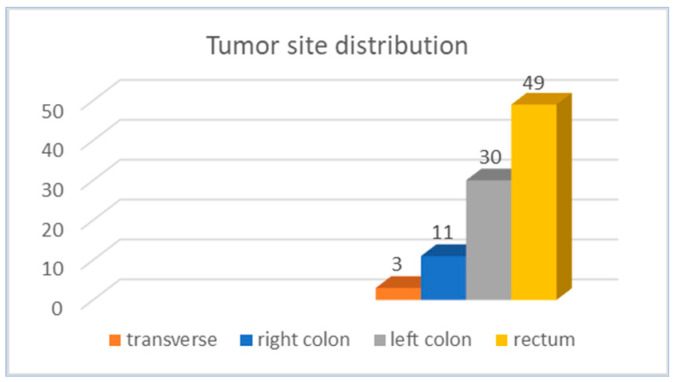
Distribution of patients with EO-CRC by tumor site.

As for tumor location, to simplify the analysis, four main colorectal regions were illustrated: the right colon, including the cecum, ascending colon, and hepatic flexure; the transverse colon; the left colon, comprising the splenic flexure, descending colon, and sigmoid; and the fourth region, which included the rectosigmoid junction and rectum. The majority of the tumors (84.9%) were identified in the rectum and left colon (Figure 4).

**Figure 5:**
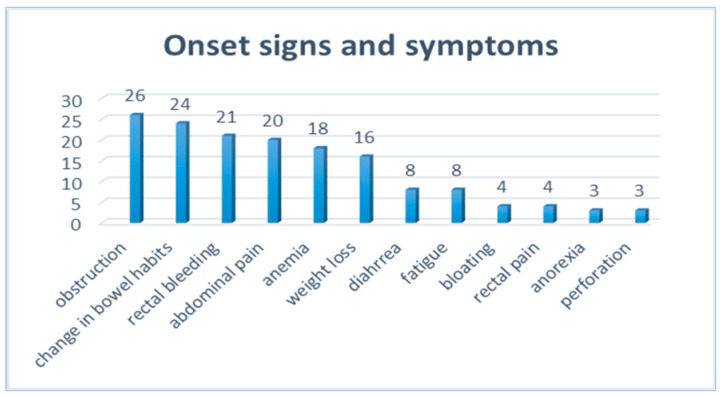
Representation of the onset signs and symptoms among EO-CRC patients in the study.

The predominant clinical presentation was intestinal obstruction, which occurred in 26 cases, followed by a change in bowel habits and rectal bleeding. Also, most of the patients presented with more than one symptom at admission. The variety of signs and symptoms that characterized the initial clinical picture in our patients is illustrated in Figure 5.

Nearly half of the patients presented to the emergency department unit (43% of the cases), and for the rest of the patients, a programmed admission was possible. More than half of the cases (59.1%) presented with complicated tumors, with obstruction being the main type of complication (50.9%). In the majority of the cases (69.9%), surgical procedures with curative intent were performed, whereas in 14 cases the surgical attitude included palliative interventions. The associated pathological conditions included obesity in most of the cases (12 patients), metabolic syndrome in 5 cases and diabetes in 4 patients. One case with Turner syndrome was identified. When analyzing the data from the pathological report, adenocarcinoma was the predominant histological type in 80.6% of the cases. The main histological subtype was classical adenocarcinoma, in more than half of the cases, and the majority of the tumors exhibited medium-grade differentiation (grade 2, G2). In 12.9% of the cases, synchronous polyps were identified on the pathological specimen, showing a histology of adenoma with various grades of dysplasia or an aspect of hyperplastic polyp. Except for one case, where diffuse polyposis was diagnosed and total colectomy was performed, all the cases involved less than ten polyps. All the patients with polypoid lesions were in the 40–49 age group. Most of the cases presented with advanced disease, having locoregional or distant spread; colorectal cancer cases with locoregional extent (stage III) were predominant. In 20 patients, distant metastases were present, with the main site of the distant lesions being the liver (18 cases), followed by the lung (5 cases), peritoneal carcinomatosis in 3 cases, and bone metastasis in 3 cases. In one case only, brain and kidney metastases were present. Some of the patients developed more than one site of metastatic disease, presenting with a combination of two or three metastatic regions.

Synthetic data regarding demographic features, clinicopathological, and therapeutic aspects of the study group are presented in Table 1.

## 4. Discussion

In recent decades, there has been a decline in the incidence and mortality of CRC overall; however, current data indicates a rise in its occurrence among individuals under the age of 50 [21]. The initial observation of an increasing occurrence of early-onset colorectal cancer was documented within the population in 2003 [22].

The majority of cases occur sporadically, while only a minority of cases represent hereditary genetic syndromes; of these, familial adenomatous polyposis (FAP) and Lynch syndrome (LS) are the most common [21].

In a paper from 2005, Wild introduces the term exposome, defined as the entire lifetime exposure to environmental factors, from conception onwards [23]. Regarding early-onset colorectal cancers, the vast majority of the cases are supposed to be driven by exposomal elements, independent of genetic determinism [24]. Obesity during adolescence has been associated with increased occurrence of EO-CRC [22,25]. Other suggested factors include diabetes mellitus, dietary patterns, the coexistence of metabolic syndrome, and a sedentary lifestyle, yet they fail to entirely explain the rise in EO-CRC [26,27]. Obesity was the most frequently associated condition among the population in the study, accounting for nearly 13% of the cases; in 5.3% of the cases, a metabolic syndrome was identified.

Substantial evidence points towards the role of human microbiota in cancer development and progression [28], in the way that carcinogenesis may be influenced by altered microbiota via several mechanisms such as modulation of inflammation, DNA damage, or the production of a variety of metabolites involved in cancer promotion or tumor suppression [29,30]. In particular, for colorectal cancer, there is increasing evidence to support the role of microbiota in tumorigenesis [31], with specific bacterial species being incriminated (such as Fusobacterium nucleatum, enterotoxigenic Bacteroides fragilis, and colibactin-producing Escherichia coli), alongside overall dysbiosis [10,31]. Early-life exposures and subsequent lifestyle factors directly influence the gut microbiome. Exploring the interaction between these exposures, the gut microbiome, and early-onset colorectal cancer is challenging and further data collection is needed [10,32].

Across the seven-year study period, the total number of patients with colorectal cancer admitted each year increased, except during the years 2020 and 2021 when a downward trend was noticed. Nonetheless, the incidence of EO-CRC cases was highest in 2020 and 2021. The lowest number of patients was noticed in 2020, which is the year when the COVID-19 pandemic was declared, with the decreased referral being caused by the restrictions imposed in that epidemiological context. The COVID-19 pandemic had a negative impact on patients’ referrals, diagnoses and treatments for colorectal cancer, as observed in several other studies [33,34].

In the present study, the vast majority of the patients belonged to the 41–49 years group (78.4%). This is consistent with the findings of You et al. who, in addition, showed a median age for young-onset CRC of 44 years, which is similar to the median age in our study [9].

Regarding gender distribution, studies have shown an equal distribution for both sexes [21] or a slightly male predominance [35], as described in our study.

Regarding clinicopathological characteristics, several studies have shown that individuals with EO-CRC possess distinctive tumor site locations, histologic features, and presentation stages [11].

Patients with colorectal cancer diagnosed before the age of 50 tend to present in advanced stages at the time of diagnosis (with regional or distant disease). This subset of patients receives more aggressive therapies, resulting in extended overall 5-year survival [36]. Survival rates are lower for patients under 30 years old, whereas they are similar or even superior for patients aged between 40 and 50 when compared to those over 50 years old [4]. A valid explanation for this survival rate stratification cannot be formulated based on the present study, but existing data speculate the role of a distinct biological background in very-early-onset CRC that may trigger an earlier and faster progression [37]. Disparities in terms of survival and mortality were observed in different racial or ethnic groups [38,39,40]. The 5-year relative survival was found to be better for White patients when compared to Asian and Hispanic patients and was worse for Black patients [38,39]. The factors underlying these disparities are numerous and may include unequal access to medical care and cancer therapies, socio-cultural elements, and also genetic and biological differences as contributing determinants [41,42]. More than half of the patients in our study (50.5%) were diagnosed with advanced-stage disease, with loco-regional or metastatic spread. Late-stage disease in the young may be explained through early misdiagnoses or late presentation. A study by Scott et. al. found a median time of 217 days from the start of rectal cancer symptoms to treatment for patients under 50 years of age compared with 29.5 days in the case of patients aged 50 years or older [43].

Typical colorectal cancer symptoms—abdominal pain, fatigue, or weight loss—and the more frequent symptoms related to the left-sided localization (changes in bowel habits, rectal bleeding) may be attributed to common conditions in young patients, thus omitting a cancer suspicion and delaying diagnosis [15,44]. The main clinical presentation in our study was represented by intestinal obstruction, followed by left colon cancer symptoms (a change in bowel habits and rectal bleeding).

The rate of complicated tumors requiring acute or emergent interventions was found to be around 33% in patients diagnosed with CRC, regardless of age [45]. In our study, this rate was considerably higher—more than half of the included cases (59.1%) presented with a cancer-related complication (intestinal obstruction, intestinal bleeding, or perforation), suggesting a potentially more aggressive tumor profile in the young or a delay in presentation and diagnosis.

Furthermore, these patients require acute or emergent surgical interventions, with higher rates of morbidity and mortality compared to those treated in an elective manner [45].

Regarding the tumor site, left-sided tumors (distal and rectal tumors) are frequently encountered in younger patients [16,46]. In a retrospective cohort study conducted on over 250,000 patients diagnosed with colorectal cancer, Abdelsattar et al. showed that young patients are more likely to have rectal cancer [36,39]. Similarly, in our study, the majority of the tumors were located in the rectum (52.6%) and left colon (32.2%). Given that a significant percentage of colorectal cancers among younger patients are located in the left colon [11], the utilization of sigmoidoscopy emerges as an important tool for the assessment of rectal bleeding in these patients. If no anorectal cause of bleeding is found, a colonoscopy is advised [47].

Mucinous and signet ring cell histology, along with poorly differentiated tumors, are commonly found in young-onset colorectal cancer patients [11,47]. When analyzing the histological reports, mucinous histology was the second most frequent histology identified in the study, accounting for 18.2% of the tumors, after traditional adenocarcinoma. Compared to late-onset colorectal cancer, in EO-CRC cases, mucinous and signet ring cell histology are more frequently encountered as well as poor differentiation [11]. In our study, most of the tumors presented medium differentiation, followed by poorly differentiated tumors.

In a study using the SEER national cancer database, O’Connell et al. found a higher prevalence of mucinous and signet cell tumors in younger patients compared to those aged 60 or older [48]. The presence of mucinous histology correlates with a higher occurrence of microsatellite instability (MSI-H), a factor that is commonly associated with Lynch syndrome and sporadic colorectal cancer [49,50]. Lynch syndrome patients with MSI/dMMR exhibit a good prognosis, while in sporadic colorectal cancer tumors, which are more frequently right-sided, the prognosis varies with the tumor stage [50]. Although recommendable, the MMR status was not available in the present study.

Another finding in our study was the incidence of polyps in the studied cases—12.9% of the patients presented with colorectal polyps, which were identified at the moment of cancer diagnosis or in the follow-up period. The most frequent type was adenomatous polyps. In addition, in the present study, both synchronous and metachronous tumors were identified, accounting for 6.4% of the cases. Colorectal cancer in the young is associated with a higher incidence of synchronous and metachronous tumors and correlates with a greater likelihood of developing polyps during the follow-up period [51]. When comparing clinicopathological and molecular biological features between patients younger than 40 years with those older than 60 years, Liang et al. identified an increased incidence of synchronous and metachronous colorectal cancer for younger patients. This may impact clinical attitude towards more vigilant preoperative and postoperative monitoring [17].

Obtaining a degree of control over the rising incidence of colorectal cancer in the young population might be achieved through the implementation of specific measures on various fronts. Synthetically, these could be divided into several levels, including etiological research, educational measures regarding this pathology, screening programs, and the implementation of specialized clinical care centers and also running clinical trials with a focus on EO-CRC [4,10,21]. To better understand the etiology of EO-CRC, complex and multidirectional etiological research is needed. This may include life-course epidemiological studies combined with genetic risk factors analysis and omics techniques (genomic, metagenomic, and metabolomic studies). All these strategies are reunited under the umbrella term of molecular pathological epidemiology (MPE) [10]. This new field enriches life-course epidemiology with a biological fundament and states that the development of a disease arises from the cumulative effect of genetic and epigenetic changes as well as the interaction between affected cells and both internal and environmental factors [52].

Another level is directed towards educational measures, for both healthcare providers and the general population. The general population might be trained in terms of changing modifiable lifestyle factors that are putative for CRC etiology but also in the direction of seeking medical care in the presence of common CRC symptoms (rectal bleeding, a change in bowel habits, abdominal pain) [10,47].

Screening effectiveness has been well-established in colorectal cancer in terms of reducing cancer mortality in average-risk men and women [12]. The majority of screening programs around the world are based on fecal occult blood tests, the most common strategy being the fecal immunochemical test (FIT) due to its high specificity, reliable sensitivity, and affordability [53].

Due to the limited performance of current screening strategies in diagnosing EO-CRC, new biomarkers are needed [54]. Standard colonoscopy has its disadvantages because it is an invasive procedure with some potential risks and bears significant costs in case of its implementation in the average-risk population. The non-invasive methods to detect CRC do not possess absolute sensitivity; therefore, new biomarkers are needed to facilitate the detection of patients with EO-CRC.

Although tissue biopsy remains the gold standard for cancer diagnosis, liquid biopsy has emerged in the past decades as a promising tool for the detection of significant biomarkers with clinical and therapeutical implications. A liquid biopsy approach is particularly suited in colorectal cancer due to its high rate of shedding circulating tumor fragments (DNA, cells, and methylation markers) [55]. Of these, circulating tumor DNA (ctDNA) represents a potentially reliable biomarker in CRC, exhibiting a broad utility that encompasses the early diagnosis and molecular profiling, the detection of minimal residual disease, monitoring for early recurrence, and assessing the acquired resistance mechanisms [55,56,57]. Although it is very attractive through its ease of performance and availability, this method might present important drawbacks from the standpoint of sensitivity and specificity in early cancer detection, as there are findings supporting that in early and asymptomatic cases, an insufficient amount of ctDNA exists in the bloodstream to yield an accurate test result [58]. Nonetheless, there are several ongoing trials that are investigating the role of ctDNA as a potential marker for minimal residual disease assessment (TRACC, NCT04050345; ADNCirc, NCT02813928) and also other trials that are assessing the role of ctDNA in identifying patients with stage II colon cancer with a high risk of cancer recurrence (COBRA, NCT0406810; CIRCULATE, NCT04120701; DYNAMIC- II, ACTRN12615000381583) [56].

Nakamura et al. investigated a novel liquid biopsy assay that could have the potential to be used with high accuracy in the early detection of patients with EO-CRC. These biomarkers included microRNA signatures, which were identified in plasma specimens from patients with young-onset colorectal cancer [54].

In 2021, the US Preventive Services Task Force (USPSTF) updated the recommendations regarding the starting age for colorectal cancer screening, reducing it to 45 years in individuals with an average risk for colorectal cancer. This measure is justifiable considering that the majority of patients in the early-onset group are in the 45–50 years interval, as shown in the present report and in other studies [9]. Nevertheless, this shift in attitude would translate into a moderate elevation in life years gained and a reduction in both colorectal cancer incidence and mortalities when contrasted with the initiation of screening at 50 years of age [13,59]. Nationwide, some pilot colorectal cancer screening programs have been established, but the Romanian public health policies are deficient in terms of a coherent and unified colorectal cancer screening program.

The constraints of our research encompassed insufficient data concerning colorectal cancer risk factors, colorectal cancer screening records, familial medical histories, hereditary cancer conditions, or the presence of chronic inflammatory colitis. The present study included cases from a singular institution and examined a limited sample size, for which statistical analysis was deemed inadequate. Further studies are warranted to include multiple centers nationwide for specific periods of time to enhance the conclusions about national trends regarding this pathology in the young.

For the present study, molecular data were available only in isolated cases and a solid conclusion could not be formulated. Therefore, future studies including validated biomarkers in CRC are needed to enhance the knowledge of the molecular mechanisms underlying early-onset colorectal cancers.

## 5. Conclusions

The increase in colorectal cancer among younger patients, which has led to a subsequent rise in mortality in recent decades, is well documented.

The characteristics of the early-onset colorectal cancer patient population analyzed in the present study largely overlap with the classic clinical, pathological, and therapeutic features highlighted in the literature for this clinical entity.

Diagnosing patients in advanced stages of the disease requires a prompt diagnostic approach regarding alarming digestive symptoms in patients under 50 years old.

The fact that we detected a significant percentage of patients within the study in the age group of 45–49 years necessitates reconsideration of the starting age threshold for colorectal cancer screening in young adults with an average risk of the disease.

Furthermore, formulating a precise diagnosis for young patients with colorectal cancer requires integrating genetic and molecular biology data, which will serve as prognostic and predictive elements.

## Figures and Tables

**Table 1:** Demographic and clinicopathological features of EO-CRC study group.

Feature	Colon Cancer (*n* = 44)	Rectal Cancer (*n* = 49)	Overall (*n* = 93)
Gender (%) ▪ Male ▪ Female			
	20 (45.4)	30 (61.2)	50 (53.8)
	24 (54.5)	19 (38.8)	43 (46.2)
Age, years (mean ± SD) Age range; median	42.3 ± 5.29	43.9 ± 4.70	43.21 ± 5.03
	24–49; 43.5	28–49; 45	24–49; 45
Area of origin (rural vs. urban)	22; 22 (50; 50)	26; 23 (53.1; 46.9)	48 (51.6); 45 (48.4)
Type of admission ▪ Emergency department (ED) ▪ Non-ED			
	23 (52.3)	17 (34.7)	40 (43)
	21 (47.7)	32 (65.3)	53 (57)
Cases with complication (no., %) ▪ Obstruction ▪ GI bleeding ▪ Perforation	26 (59)	29 (59.1)	55 (59.1)
	15 (34.1)	13 (26.5)	28 (30.1)
	7 (15.9)	16 (32.6)	23 (24.7)
	4 (9.1)	0	4 (4.3)
Type of surgery (%) ▪ Radical ▪ Palliative ▪ Biopsy ▪ None			
	36 (81.8)	29 (59.1)	65 (69.9)
	5 (11.4)	9 (18.4)	14 (15)
	2 (4.6)	4 (8.1)	6 (6.5)
	1 (2.2)	7 (14.3)	8 (8.6)
Presence of polyps	4 (9)	8 (16.3)	12 (12.9)
Metachronous/Synchronous tumors	3/1	0/2	3 (3.2)/3 (3.2)
HP type (%) ▪ Adenocarcinoma (ADK) ▪ Squamous carcinoma ▪ Carcinoma with sarcomatoid components ▪ GIST			
	39 (88.6)	36 (73.4)	75 (80.6)
	0	1 (2)	1 (1)
	0	1 (2)	1 (1)
	1 (2.2)		1 (1)
HP subtypes (%) ▪ Adenoma-like ADK ▪ Classical ADK ▪ Micropapillary ADK ▪ Mucinous ADK ▪ Signet ring cell ADK			
	0	2 (4)	2 (2.15)
	25 (56.8)	23 (46.9)	48 (51.6)
	0	1 (2)	1 (1)
	11 (25)	6 (12.2)	17 (18.2)
	1 (2.2)	1 (2)	2 (2.1)
Tumor grade (%) ▪ G1 ▪ G2 ▪ G3			
	9 (20.4)	6 (12.2)	15 (16.1)
	16 (36.3)	21 (42.8)	37 (39.7)
	11 (25)	4 (8.1)	15 (16.1)
(y)pTNM (%) ▪ 0–I ▪ II ▪ III ▪ IV			
	2 (4.5)	9 (18.3)	11 (11.8)
	10 (22.7)	8 (16.3)	18 (19.3)
	16 (36.4)	11 (22.4)	27 (29)
	13 (29.5)	7 (14.2)	20 (21.5)

## Data Availability

The data presented in this study are available upon request from the corresponding author.
